# Differences in Physiological Reactions Due to a Competitive Rehabilitation Game Modality

**DOI:** 10.3390/s21113681

**Published:** 2021-05-25

**Authors:** José M. Catalán, José V. García-Pérez, Andrea Blanco, David Martínez, Luis D. Lledó, Nicolás García-Aracil

**Affiliations:** Biomedical Neuroengineering Research Group of the Bioengineering Institute, Miguel Hernandez University, 03202 Elche, Spain; j.garciap@umh.es (J.V.G.-P.); ablanco@umh.es (A.B.); david.martinezp@umh.es (D.M.); llledo@umh.es (L.D.L.); nicolas.garcia@umh.es (N.G.-A.)

**Keywords:** rehabilitation, multiplayer games, interpersonal rehabilitation games, patient engagement, exergames, robotics

## Abstract

Interpersonal rehabilitation games, compared to single-player games, enhance motivation and intensity level. Usually, it is complicated to restrict the use of the system to pairs of impaired patients who have a similar skill level. Thus, such games must be dynamically adapted. Difficulty-adaptation algorithms are usually based only on performance parameters. In this way, the patient’s condition cannot be considered when adapting the game. Introducing physiological reactions could help to improve decision-making. However, it is difficult to control how social interaction influences physiological reactions, making it difficult to interpret physiological responses. This article aimed to explore the changes in physiological responses due to the social interaction of a competitive game modality. This pilot study involved ten unimpaired participants (five pairs). We defined different therapy sessions: (i) a session without a competitor; (ii) two sessions with a virtual competitor with different difficulty levels; (iii) a competitive game. Results showed a difference in the physiological response in the competitive mode concerning single-player mode only due to the interpersonal game modality. In addition, feedback from participants suggested that it was necessary to keep a certain difficulty level to make the activity more challenging, and therefore be more engaging and rewarding.

## 1. Introduction

In the last report from the Stroke Alliance for Europe [1], around 15 million people worldwide suffer from stroke every year. Through the data collected by the Global Burden of Disease study in 2015 and demographic projections obtained from Eurostat (statistical office of the European Union, EU), the number of strokes is expected to rise to 34% between 2015 and 2035 in the EU. With the welcome improvements in the survival rate, the number of post-stroke people has increased, which increases the need for care and rehabilitation. In 2015, the EU dealt with an estimated combined direct and indirect cost of €45 billion. Reducing the incidence of stroke and the likelihood of long-term disability will help to reduce these costs [2,3].

The effectiveness of rehabilitation in improving functioning and quality of life is higher in a high-intensity, reproducible therapy. Professionals have also described motivation as an important determinant of rehabilitation outcome [4]. It has been shown that multicenter clinical trials with robots can achieve long-term results comparable to exercise with a therapist [5,6]. In addition, rehabilitation robotic devices have shown promising results to increase rehabilitation therapy intensity and motivation [7,8,9].

A promising way to maintain a high level of motivation during long-term rehabilitation therapies is to use interpersonal rehabilitation games in which patients cooperate or compete in a game. Several studies demonstrated that interpersonal rehabilitation games obtain better results in increasing motivation and exercise intensity compared to conventional rehabilitation exercises [10,11,12,13].

One of the main research topics on interpersonal rehabilitation games is the development of difficulty-adaptation methods. To obtain good outcomes in a competitive or cooperative rehabilitation game, patients should be on an equal footing to ensure a proper level of competitiveness. Usually, it is complicated to limit the use of the system to pairs of impaired patients who have a similar skill level. That is why the system should be adapted to the condition of the patient.

In competitive and cooperative rehabilitation games, the difficulty-adaptation methods are usually based on performance parameters [14,15]. Evaluating the patient’s condition and adjusting the therapy accordingly comprise a complex problem. By controlling the therapy conditions, it is possible to modulate the system as a function of the patient’s state [16,17]. However, an interpersonal rehabilitation game is an even more complex paradigm since social interaction could affect the patients’ physiological response during the exercise. That makes it challenging to determine the different cognitive and affective states of the patients. Currently, new methods are underway to adapt the exercises considering the patient’s condition [18,19]. However, it is difficult to discern whether the physiological reactions are due to the game’s difficulty or social interaction. This aspect is key to quantifying the patient’s capabilities and determining whether to increase or decrease the game’s difficulty.

This article aimed to study how the user’s physiological reactions are affected due to the interpersonal game modality. To perform this, we defined a single-player game modality with a virtual competitor with an intensity level very similar to the one of the competitive mode. In addition, we limited the social interactions during the competitive mode, preventing users from communicating with each other during the game. In this way, we measured differences in the physiological responses of the users only due to the type of competitor. Moreover, we also studied two single-player game modes, one without a competitor and another with an easy difficulty level. These served as a reference when studying the levels of motivation and intensity.

## 2. Methods

### 2.1. Subjects

A total of 10 subjects (9 men, 1 woman) with no motor or cognitive impairment were recruited for the study. They were between 23 and 50 years old (31.6 ± 9.5 years). All of them were right-handed. They were recruited from the staff of the Bioengineering Institute of Miguel Hernández University.

Pairs of participants were recruited according to approximate age, gender, and handedness (except in one case, in which a woman competed with a man). In two-player game studies, it is common to match by gender [12,20] because significant differences in game experience have been found due to gender [21]. Similarly, significant differences in game experience have been found between young and old players [22]. In addition, both members of a pair were already familiar with each other to some degree. Familiarization with rehabilitation robots was considered as an exclusion criterion.

### 2.2. Experimental Setup

**Figure 1:**
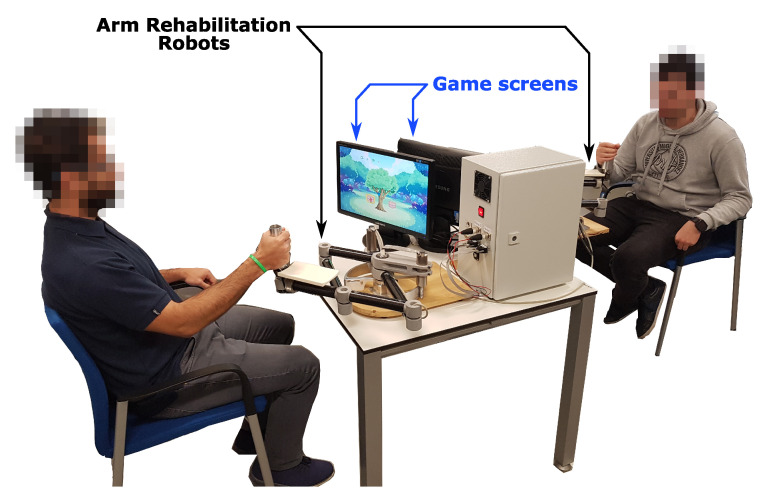
Overview of the experimental setup with two participants.

Figure 1 shows an overview of the experimental setup used in this study. Each participant sat in front of a robot and grasped the end-effector with his/her dominant hand. Two screens placed in front of each subject displayed the game. They were facing each other. They could not see each other and were instructed not to interact during the game session. However, they were allowed to interact between conditions.

### 2.3. Game

**Figure 2:**
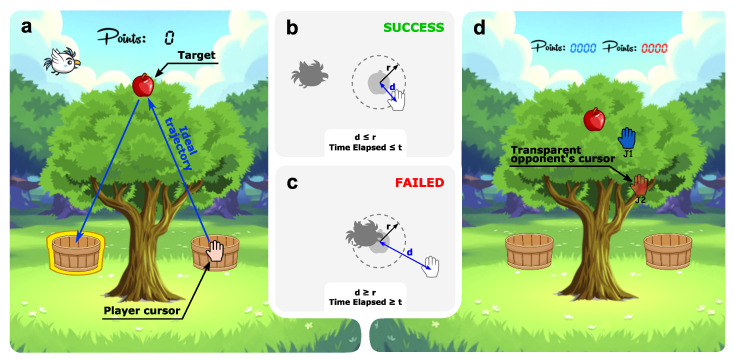
Overview of the game. (**a**) A screenshot of the single-player game mode. (**b**) Condition of successfully reaching the target, where *d* is the distance of the player cursor to the target, *r* is the minimum distance to reach the target successfully, and *t* is the set time to reach the target. (**c**) Condition of failing to reach the target. (**d**) A screenshot of the competitive game mode.

Figure 2 shows an overview of the game. It consisted of a point-to-point modality. The player cursor was represented by a hand whose center corresponded to the actual position of the patient. A bird represented the virtual competitor.

Firstly, the player had to wait in the basket until an apple appeared (Figure 2a), then try to reach it faster than the bird and then drop the apple in the indicated basket. For each apple collected, players accumulated some points.

A target was considered reached when the distance of the player cursor to the apple, *d*, was less than or equal to the distance *r* (Figure 2b). In this study, the distance *r* was set to 1 cm for all game modes. The time *t* (Figure 2b,c) was the amount of time the user had to reach the target before the bird reached it.

The basket, in which the user had to drop the apple, was chosen randomly while guaranteeing both baskets were reached an equal amount of times.

There were two game modalities:

- A single-player game modality (Figure 2a): In this modality, participants took a certain number of apples freely or competed against the bird. In the latter case, the difficulty level was adjusted by setting the *t* parameter. The goal consisted of scoring as many points as possible;
- A multiplayer game modality: This modality consisted of a competitive game where two players participated simultaneously (Figure 2d). Participants played against each other and tried to obtain more points than the other. Points were assigned according to the order of arrival at the basket, so participants had to take the apple and leave it in the basket before their competitor. During the game, players could see the score and the position of the other player.

### 2.4. Arm Rehabilitation Robot

In this study, two identical robotic devices for upper limb rehabilitation were used [23,24]. This rehabilitation platform consisted of a robotic system with two actuated degrees of freedom. It was designed to be placed on a table and used sitting on a chair.

Since the participants were unimpaired, the robotic platform did not provide any assistance or compensation.

### 2.5. Estimation of the Exercise Intensity

The velocity of the hand has been proven to be a reasonable estimation of energy consumption during arm rehabilitation therapies in post-stroke patients [25]. Therefore, it acts as an objective measure of exercise intensity estimation. We extracted the Root Mean Square (RMS) velocity value from the speed profile described by the users in every trial. The RMS velocity value of the hand is closely related to the energy expenditure during upper limb exercise, compared to estimates based on heart rate response, electromyography activity, or oxygen consumption [25,26]. We also extracted maximum and mean velocity values to have more information when studying differences in intensity levels among the different game modes.

In addition, we measured the reaction time from when the apple visually appeared until the player started to move towards it.

### 2.6. Estimation of Exercise Performance

The score of a game is used extensively as a measure of exercise performance [18,27]. It allowed us to evaluate whether or not the participants could achieve the objective of the game. In other words, it was representative of the difficulty level of the game.

### 2.7. Measurement of the Physiological Response

Two Shimmer3 GSR+ sensor units were used, one for each subject. This device has a built-in signal-processing unit that sends the resulting information to the central processing unit via Bluetooth. The output measure is the Galvanic Skin Response (GSR) between two reusable electrodes placed on two fingers of the hand. GSR is a standard measure in psychophysiological paradigms and, therefore, often used in affective state detection. In this study, we placed the electrodes on the proximal phalanges of the index and middle finger of the hand not used to control the robot (non-dominant hand). The sample rate of the sensor unit was 50 Hz. From the GSR, we extracted the Skin Conductance Response (SCR).

We also recorded the Electrocardiogram (ECG) of each participant through the Zephyr BioHarness^TM^ (Zephyr Technology Corporation) physiological monitoring telemetry device. The BioHarness transmits signals to be received via Bluetooth. This device also has a built-in signal-processing unit, so the received signal is already processed. The sampling rate of the sensor unit was 250 Hz. We extracted the Heart Rate (HR) evolution over each condition from the ECG signal. HR measurement is standard in rehabilitation games [8].

To study how the condition affected the user’s affective state, we obtained the value reached at the end of every condition for each physiological feature. We normalized all the physiological features by the min-max normalization method (1).

(1) $$x_{norm}=\frac{x-x_{baseline}}{x_{max}-x_{baseline}}$$

### 2.8. Subjective Assessment of the Experience

There are currently several evaluation tools available for assessing patient motivation and satisfaction during technology-assisted rehabilitation. One of the instruments most used is the Intrinsic Motivation Inventory (IMI) [28]. This subjective questionnaire measures four aspects of engagement: enjoyment/interest, effort/importance, perceived competence, and pressure/tension. While there are many versions of the IMI [29], we decided to use a reduced version that has already been employed in other studies [10,14,30].

### 2.9. Study Protocol

**Figure 3:**
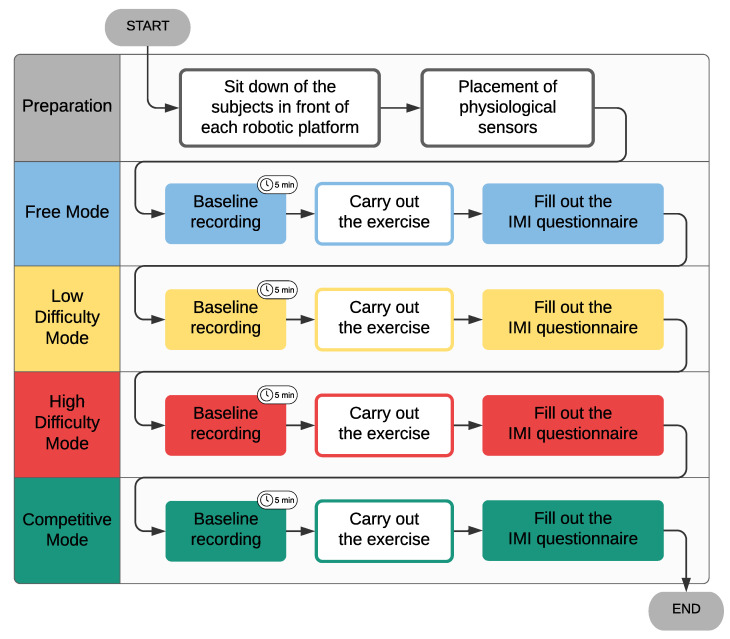
Diagram of the study protocol. The conditions were performed sequentially. Before each condition, a baseline recording period of 5 min was carried out.

Figure 3 shows the study protocol diagram. Firstly, we explained the purpose and procedure of the study to the participants. If they agreed to perform the experimental session, they were fitted with a Zephyr BioHarness^TM^ on the chest and a Shimmer3 GSR sensor unit placed on the non-dominant hand. Finally, they sat in front of the robot.

Two instances of the game were executed, one on each of the rehabilitation platforms and one for each player.

Each participant performed the different single-player game modes in the same order (Figure 3):

1. Free mode: In this mode, there was no competitor, which means that there was no time limit;
2. Low-difficulty mode: The time *t* was set as 2 s. In this case, players had a virtual competitor;
3. High-difficulty mode: In this game mode, it was challenging for the player to beat the virtual competitor since the *t* parameter was set as 0.7 s.

Each single-player game mode was performed simultaneously and separately. In this way, both participants were ready at the same time to perform the competitive mode at the end.

It is important to note that no difficulty level was configured in the free mode and the competitive mode. Therefore, the intensity level was only self-imposed by the participants.

For this study, it was decided not to randomize the conditions due to the small sample.

The number of apples was set to 21 in all cases to prevent a tie in the competitive mode. The robotic rehabilitation platform allowed us to configure the range of motion to match it with the patient’s range of movement. In this study, subjects did not have any motor or cognitive impairment, so the range of movement was set to a 10 cm diameter circle, so they all traveled the same distances.

In the protocol, the duration of each game mode was not determined. The number of points a subject earned per apple was not influenced by the amount of time required to catch it. Therefore, the only goal of the participant was to be able to catch the 21 apples.

Before each session, there was a 5-min rest period where the physiological signals were recorded to compute the baseline (Figure 3). This period was employed to relax the subject so that the previous condition did not affect the next one.

### 2.10. Statistical Data Analysis

In the statistical study, we carried out a normality test through the Shapiro–Wilk test. There is evidence to suggest that some parameters were not normally distributed.

For the purposes of data analysis, all 10 participants were treated as independent.

One-way repeated-measures Analysis of Variance (ANOVA) was employed for normally distributed parameters. We used Mauchly’s test of sphericity to evaluate whether the sphericity assumption was violated. If sphericity was violated, repeated-measures ANOVA was corrected using Greenhouse–Geisser correction when epsilon was ϵ<=0.75 or Huynh–Feldt correction when epsilon was ϵ>0.75. In the post hoc analysis, the assumption of equal variances across groups (homoscedasticity or homogeneity of variances) was studied with Bartlett’s test. Tukey post hoc tests or Games–Howell post hoc tests were used depending on Bartlett’s test result.

On the other hand, the Friedman test was used for not normally distributed parameters. In the post hoc analysis, the Holm–Bonferroni method was used to adjust for familywise error rate correction.

Finally, the magnitude of Spearman’s rank correlation coefficient ($r_{s}$) was obtained to study how one variable affected another.

## 3. Results

### 3.1. Exercise Intensity

**Figure 4:**
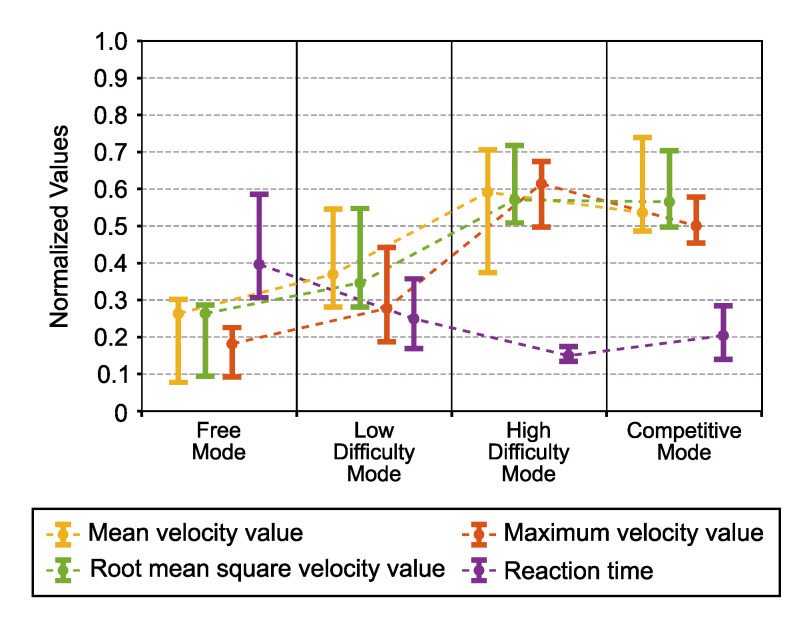
Exercise intensity parameters. All parameters were normalized by the min-max normalization method and are graphically represented in each game mode by the median value and an error bar representing the first and third quartiles.

In Figure 4, the parameters directly related to the exercise intensity are shown.

The root mean squared velocity value showed significant differences between conditions (one-way repeated-measures ANOVA p=0.0001). Paired comparisons showed that all single-player modes were significantly different, except in the free mode concerning the low-difficulty mode (p=0.32). Regarding competitive mode, we obtained a statistically significant difference concerning free mode (p=0.0002) and the low-difficulty mode (p=0.027) but not in the high-difficulty mode, which suggested that the intensity level was almost the same.

We also obtained similar results for the case of the mean velocity value.

The analysis also showed significant differences between the game modes for the maximum velocity value (Friedman test p<0.0001). In the paired comparisons, we obtained that all modes were significantly different (p<0.007), even the high-difficulty mode with respect to the competitive mode (p=0.047).

Regarding the reaction time, analysis also showed significant differences between conditions (Friedman test p=0.002). The low-difficulty mode had a barely statistical significant difference with respect to the high-difficulty mode (p=0.05), but was not significantly different from the competitive mode (p=0.29). Furthermore, a difference between the competitive mode and high-difficulty mode narrowly eluded statistical significance (p=0.085). In the post hoc analysis, we found a moderate correlation between the reaction time and root mean squared velocity value (rs=−0.66, p<0.001).

### 3.2. Exercise Performance

**Figure 5:**
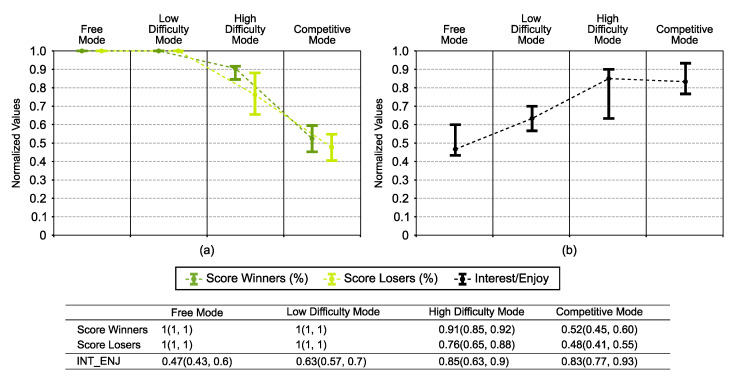
Representation of the parameters related to exercise performance. (**a**) Graphical representation of the game score, divided between winners and losers of the competitive mode. (**b**) Graphical representation of the interest/enjoy parameter from the Intrinsic Motivation Inventory. All parameters were normalized by the min-max normalization method and are graphically represented in each game mode by the median value and an error bar representing the first and third quartiles. The table collects the normalized values of all parameters.

Figure 5 contains several graphs that show the results of the parameters related to exercise performance.

In the result of the score (Figure 5a), we saw that the value decreased with increasing difficulty level.

In the competitive mode, the participants’ score was around 50%, which means that couples were well defined since there was not much difference between winners and losers, so it was a close game.

On the other hand, the results showed that the interest/enjoy parameter from the IMI increased with the difficulty level, reaching the highest values in the high-difficulty mode and the competitive mode.

Post hoc analysis suggested that the interest/enjoy parameter was weakly correlated with the root mean squared velocity value (rs=0.37, p=0.003).

### 3.3. Physiological Reaction

**Figure 6:**
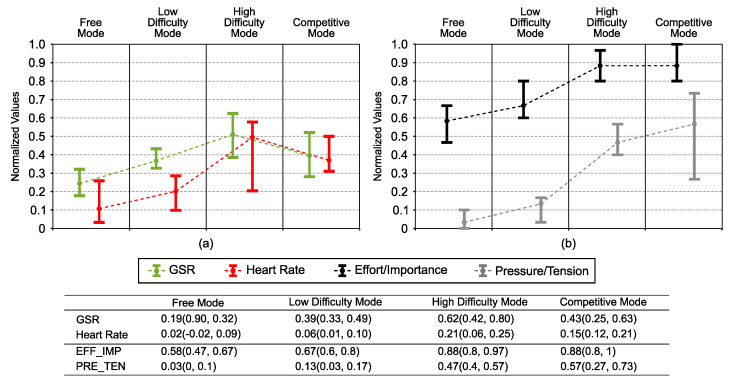
Representation of the results of the parameters related to the user’s affective state. (**a**) Results of the Galvanic Skin Response (GSR) and the heart rate. (**b**) Results of the pressure/tension and effort/importance parameters from Intrinsic Motivation Inventory. All parameters were normalized by the min-max normalization method and are graphically represented in each game mode by the median value and an error bar representing the first and third quartiles. The table collects the non-normalized values of all parameters.

Physiological response signals are illustrated graphically in Figure 6a,b. Parameters from the IMI related to the user’s affective state are also illustrated in Figure 6.

GSR (Figure 6a) showed significant differences among groups (one-way repeated-measures ANOVA p=0.005). However, in the pairwise comparison, no significantly different pairs were found. However, results showed an upward trend with increasing intensity level, although a lower value than in the high-difficulty level was observed in the competitive mode.

Post hoc analysis reflected a weak correlation between the GSR and the score (rs=−0.29, p=0.016). Results also suggested that GSR was almost weakly correlated with the root mean squared velocity value (rs=0.28, p=0.051) and the reaction time (rs=−0.18, p=0.08). We also observed a reliable trend toward significance in the case of the correlation between GSR and the interest/enjoy parameter from the IMI (rs=0.28, p=0.051).

Regarding HR (Figure 6a), results showed differences between game modes (one-way repeated-measures ANOVA p=0.0001). As we observed for GSR, the results indicated that HR increased with increasing intensity. However, in the competitive mode, we also observed a lower value than the high-difficulty mode.

Post hoc analysis suggested that HR was moderately correlated with the root mean squared velocity value (rs=0.55, p<0.001) and the reaction time (rs=−0.54, p<0.001). We also observe that HR was moderately correlated with the score (rs=−0.47, p=0.004). On the other hand, HR was weakly correlated with the interest/enjoy parameter from the IMI (rs=0.27, p=0.017).

In Figure 6, parameters from the IMI related to the user’s affective state are shown. We observed that the value increased when increasing the intensity level for both the pressure/tension parameter and the effort/importance parameter, reaching the highest values in the high-difficulty mode and the competitive mode.

In the post hoc analysis, a strong correlation with the root mean squared velocity value was found with pressure/tension (rs=0.72, p<0.001) and a moderate correlation with effort/importance (rs=0.43, p=0.01). This was also the case for the reaction time (rs=−0.4, p=0.001 and rs=−0.21, p=0.026, respectively). Results also suggested that both were weakly correlated with the score and the interest/enjoy parameter.

We also note that effort/importance was strongly correlated with GSR (rs=0.68, p<0.001) and presented a weak correlation with a statistical trend towards significance with respect to HR (rs=0.2, p=0.098). In the case of the pressure/tension, this parameter was moderately correlated with HR (rs=0.51, p=0.001) and GSR (rs=0.45, p=0.014).

## 4. Discussion

The differences between conditions were largely due to the defining of the different game modes. Therefore, although importance was given to statistically significant differences among groups, in the discussion of the results, special attention was given to the trend of the parameters among modes, although these were not statistically significant.

### 4.1. Differences in the Exercise Intensity

As previously mentioned, the intensity level was estimated mainly through the root mean squared velocity value. Firstly, we observed that modes in which an intensity level was established were significantly different (Figure 4). That means that difficulty levels were well defined. Although we did not obtain a statistically significant difference regarding free mode, results showed that the intensity level was the lowest. On the other hand, results suggested that the intensity level was broadly similar to that of the high-difficulty mode for the competitive mode. However, we observed that in the high-difficulty mode, the maximum speed reached significantly higher values. The increase in intensity level due to competition has already been widely demonstrated [10,31], also in related fields [30], so this finding was expected.

Another parameter directly related to the intensity level is the reaction time. In Figure 4, we can see how this parameter decreased as the intensity level increased.

Comparing the high-difficulty mode and the competitive mode, although we did not obtain a statistically significant difference, despite both modes having an equal intensity level, results suggested that the reaction time was longer in the competitive mode. However, this effect was not due to a different intensity level, but rather due to the game mode. We observed that as the participants could see each other inside the game, influenced how they reacted when a target appeared. In single-player modes, where the competitor was virtual, participants learned when the apple would appear, so they reacted faster. In competitive mode, the target appeared at the same time as in single-player modes, so prior learning should have made them react at least as fast as in the high-difficulty mode. However, seeing the opponent within the game made the participants look at his/her reaction, making them react slower.

Based on these results, we can conclude that despite some differences due to the game modality, the intensity levels of the high-difficulty mode and the competitive mode were very similar.

### 4.2. Evaluation of the Task Performance

In the competitive mode, the participants’ scores were around 50% (Figure 5), which means that couples were well defined since there was not much difference between winners and losers, so it was a close game.

In the high-difficulty level, the score was around 80%. To obtain the competitiveness level of the competitive mode with a virtual competitor in a single-player game, we would need to increase the difficulty level even more. However, in the high-difficulty mode, users reported that the intensity level was very high, so increasing the difficulty level could be counterproductive.

In light of the results for the interest/enjoy parameter from the IMI, participants preferred a challenging game (Figure 5b).

### 4.3. Differences in the Physiological Response

Considering that the intensity level was the same both in the high-difficulty mode and in the competitive mode, it would not be strange to obtain a similar physiological reaction in both modes. However, the GSR results suggested that the GSR value was lower than the high-difficulty level in the competitive mode. We could also observe a similar effect in the HR results. Therefore, it was a measurable effect through different physiological features.

Regarding the effort/importance parameter from the IMI, results suggested that the participants’ perceived exertion had a similar behavior as the intensity levels (Figure 4). In addition, the results indicated that this was correlated to the root mean squared velocity value and the reaction time. This means that participants were aware of the intensity level.

This finding agreed with the notion that the observed difference in the physiological reactions between the competitive mode and the high-difficulty mode was not due to the intensity level. However, this could be due to the interaction with a live player through the game.

While our results showed an appreciable difference on physiological responses, a few study limitations should be discussed. Our study involved only 10 unimpaired participants, and therefore, the results may not be generalized beyond the conditions of this study. However, the results suggested that the competitive game mode presented differences that should be validated in a more extensive study.

## 5. Conclusions

In this article, a study of how the user’s physiological reactions were affected due to the interpersonal game modality was performed. To perform this, we defined a single-player game modality with a virtual competitor with an intensity level very similar to the one of the competitive mode. In addition, we limited all social interactions, preventing users from communicating with each other during the competitive game. In this way, we measured the differences in the physiological responses of the users only due to the type of competitor.

Due to the current study design, the only difference between the high-difficulty mode and the competitive mode was the type of competitor, since we did not allow the participants to communicate with each other during the game. Knowing that they were competing with a real player seemed to modify their physiological reaction. However, we considered that this change in the physiological reaction could also be due to other aspects, such as the relationship between the two players or a more introverted personality when playing with another person, among others. Therefore, we believe that it is difficult to conclude why the physiological reaction was lower in the competitive mode. Even so, we can affirm that the results suggested that there was a difference in the physiological response concerning the single-player game mode only due to the interpersonal game modality.

In addition, it is interesting to note that according to the IMI questionnaire results, participants found the most challenging game more enjoyable. This result suggests that it is necessary to maintain a certain difficulty level to make the activity more challenging, and therefore more engaging and rewarding.
